# New Plugging Instruments

**Published:** 1868-04

**Authors:** 


### NEW PLUGGING INSTRUMENTS.

It is with pleasure that we call attention to the fine serratid plugging instruments now manufactured by S. S. White. His aim has been to make these instruments in quantities sufficient to supply the demand ; and equal in quality and perfection of form and finish to the highest execution and conception of a Palmer, Butler or Abbott. This he has so well succeeded in doing, as almost to defy criticism. We have been using these instruments for a time with a view of testing them, and we are compelled to say, that they are far superior to anything we have used before. The forms are very much improved, and the serrations most perfect. With such instruments it is a pleasure to fill teeth.

Now, when we shall have excavators and cutting instruments brought up to the same degree of perfection, it will be a happy period for the Dentist. The points in these instruments to which we hope attention will be given in the manufacture, are form, temper and fineness of finish. In regard to form, those now employed should be greatly improved, and some forms that are not in common use should be introduced. In regard to temper much remains to be attained. Every one is aware of the fact that only now and then do we find an excavator that performs well its part for any considerable length of time, and when one such is found, the wish will arise that all were so. When the idea of making these instruments for a fixed price is abandoned, and the aim be to secure perfection, regardless of price, then, and then only, may we expect what is so much desired.

We trust that the plugging instruments to which we have referred will be tested by every one who desires to make good operations. Perfect instruments lead one on to improved operations. T.

